# Implementation study of a 5-component pediatric early warning system (PEWS) in an emergency department in British Columbia, Canada, to inform provincial scale up

**DOI:** 10.1186/s12873-019-0287-5

**Published:** 2019-11-27

**Authors:** Theresa McElroy, Erik N. Swartz, Kasra Hassani, Sina Waibel, Yasmin Tuff, Catherine Marshall, Richard Chan, David Wensley, Maureen O’Donnell

**Affiliations:** 1 Child Health BC, 260 - 1770 West 7th Ave., Vancouver, BC V6J 4Y6 Canada; 2 University of British Columbia, Faculty of Medicine, 317 – 2194 Health Sciences Mall, Vancouver, BC V6T 1Z3 Canada; 3 Vancouver Coastal Health, 604-601 Broadway Ave., Vancouver, BC V5Z 4C2 Canada; 4 Provincial Health Services Authority, 200-1333 West Broadway Ave., Vancouver, BC V6H 4C1 Canada

**Keywords:** Pediatric early warning system, Pediatric early warning score, PEWS, Emergency department, Pediatrics, Implementation study, Mixed methods

## Abstract

**Background:**

The rapid identification of deterioration in the pediatric population is complex, particularly in the emergency department (ED). A comprehensive multi-faceted Pediatric Early Warning System (PEWS) might maximize early recognition of clinical deterioration and provide a structured process for the reassessment and escalation of care. The objective of the study was to evaluate the implementation fidelity, effectiveness, and utility of a 5-component PEWS implemented in the ED of an urban public general hospital in British Columbia, Canada, and to guide provincial scale up.

**Methods:**

We used a before-and-after design to evaluate the implementation fidelity, effectiveness, and utility of a 5-component PEWS (pediatric assessment flowsheet, PEWS score, situational awareness, escalation aid, and communication framework). Sources of data included patient medical records, surveys of direct care staff, and key-informant interviews. Data were analyzed using mixed-methods approaches.

**Results:**

The majority of medical records had documented PEWS scores at triage (80%) and first bedside assessment (81%), indicating that the intervention was implemented with high fidelity. The intervention was effective in increasing vital signs documentation, both at first beside assessment (84% increase) and throughout the ED stay (> 100% increase), in improving staff’s self-perceived knowledge and confidence in providing pediatric care, and self-reported communication between staff. Satisfaction levels were high with the PEWS scoring system, flowsheet, escalation aid, and to a lesser extent with the situational awareness tool and communication framework. Reasons for dissatisfaction included increased paperwork and incidence of false-positives. Overall, the majority of providers indicated that implementation of PEWS and completing a PEWS score at triage alongside the Canadian Triage and Acuity Scale (CTAS) added value to pediatric care in the ED. Results also suggest that the intervention is aligned with current practice in the ED.

**Conclusion:**

Our study shows that high-fidelity implementation of PEWS in the ED is feasible. We also show that a multi-component PEWS can be effective in improving pediatric care and be well-accepted by staff. Results and lessons learned from this pilot study are being used to scale up implementation of PEWS in ED settings across the province of British Columbia.

## Background

Globally, failure to identify and intervene with pediatric patients experiencing clinical deterioration is a source of unintended harm including prolonged hospital stays or readmission, disability and death [1–3]. Care inequities can arise due to diversity of provider experience and knowledge, and lack of standardized approaches in the management of critically ill children [1].

To maximize early recognition of clinical deterioration in children, Pediatric Early Warning Systems (PEWS) have been implemented internationally with a growing body of evidence showing benefit of their use [4–6]. PEWS should support healthcare providers in identifying abnormal physiology, tracking trends across time and supporting structured processes for reassessment and escalation of care.

In emergency department (ED) settings, the ability to identify deterioration or risk “in the moment” – without tracking over time – is critical [7]; however, rapid identification of deterioration in the pediatric population has several complexities. These difficulties include: varying normal vital signs parameters by age [8, 9], changes in physiologic parameters related to factors beyond the illness or injury (medication, pain, fear and anxiety) [10], and compensation seen in pediatric patients when critically ill, underrepresenting the degree of deterioration [7, 8]. Using PEWS in the ED could offer benefit in the focus and guidance provided on age-appropriate pediatric norms, measuring physiologic parameters and acting on evidence of abnormality [7, 11, 12].

While implementation of PEWS in EDs is becoming increasingly common globally, with mounting evidence for this application [8, 10, 11], there is still much to learn. Most studies of PEWS in ED to date focus on the predictive value of a score in determining level of care at disposition, with the overarching conclusion being that a PEWS score is reasonable at predicting admission to the intensive care unit (ICU), but has limited value in predicting admission to a non-ICU bed [10, 11, 13]. No study has yet examined PEWS as a complex healthcare intervention [4]. There has been minimal research focusing on the general utility of PEWS for the ED [10], yet utility is particularly important for ED as it is a dynamic environment with undifferentiated patients often seen for a relatively limited time [4, 10]. The operational characteristics of busy EDs include intermittent high patient loads, diagnostic uncertainty, multiple handovers, intense time pressures and diverse skills owing to high staffing levels and rapid turnover [7, 13]. Researchers, such as Roland et al. [7], built a convincing case for PEWS in ED as a means of minimizing cognitive factors and biases that impact decision making.

In British Columbia, one of the ten provinces of Canada, PEWS had not been used in ED settings prior to this study. In 2014, a provincial working group consisting of members of regional Health Authorities, academia, and healthcare providers came to a consensus that PEWS for inpatient settings should be pursued, not only to improve health outcomes but also to standardize assessment and care of children, facilitate cross-site communication processes and enhance patient transfer across the varied hospitals in the province. However, testing the effectiveness and utility of PEWS in ED settings was considered to be necessary before a provincial roll out, given a scarcity of scientific evidence.

The objective of the study was to evaluate the implementation fidelity, effectiveness, and utility of a 5-component PEWS implemented in the ED of an urban public general hospital in British Columbia (BC), Canada. This article will outline the planning, implementation, and results of this pilot study and discuss how the results are being used for provincial scale up.

## Methods

### Setting

The study setting was the ED of Richmond Hospital – an urban public general hospital located in British Columbia, Canada, with an average of 6800 pediatric visits per year; approximately 12% of total ED visits. No PEWS was in place before this study and documentation occurred on a non-pediatric specific emergency nursing assessment record. Richmond Hospital uses the Canadian Triage and Acuity Scale (CTAS) [14] to triage patients by severity and type of presenting symptoms and signs. The scale consists of five levels from 1 (resuscitation) to 5 (non-urgent).

### Intervention design

The design of PEWS for inpatients involved both an evidence review and in-depth consultation with numerous stakeholders, including Health Authority leads, quality and safety leads, expert clinicians and frontline care representatives. The result was a 5-component PEWS, consisting of the following: pediatric assessment flowsheet, Brighton PEWS score [15], situational awareness [2], escalation aid, and communication framework [16]. More detail about each component is provided in Table 1. For the pilot study of PEWS in ED, we reviewed the literature related to the ED context, studied the implementation of PEWS in the in patient setting and consulted with ED experts from around the province. The Richmond Hospital team agreed to pilot this inpatient system in the ED, understanding that tailoring to ED would be completed post pilot if the decision was made to move to scale up.

**Table 1:** Description and source of the five components that constitute the BC Pediatric Early Warning System (BC PEWS)

Component	Description	Source
Pediatric assessment flowsheet	The double-sided flowsheet, designed for inpatients, comprehensively outlines documentation for 24 h of nursing assessment, including PEWS scoring parameters, full head-to-toe assessment and documentation of routine nursing care.	Adapted from BC Children’s Hospital
	The flow sheets are available in six age grouping (0–3 months; 4–11 months; 1–3 years; 4–6 years; 7–11 years and 12+ years) to account for naturally-occurring variations vital signs norms [14]. Staff were also provided with vital signs reference cards that could be worn on a lanyard.	CTAS [14]
PEWS score	The Brighton PEWS score embedded in the flowsheet is the most widely used and validated PEWS score available for inpatient care. It is a 13-point score (with 0 normal and 13 high risk) based on behavioural, cardiovascular and respiratory parameters.	Brighton PEWS score [15]
	As the Brighton scoring tool is not age specific, vital signs references for PEWS scoring were based on the Canadian Triage Acuity Scale (CTAS) vital signs norms [14, 17]. These norms were chosen to promote internal consistency within and between sites based on a nationally accepted standard. The PEWS scoring section of the flowsheet has the norms embedded for easy plotting and is colour coded to provide a clear visual when vital signs are outside of the normal range.	CTAS [14], Fleming et al. [17]
Situational awareness	The intent of situational awareness is to promote awareness, prediction, and mitigation of potential risk. Implemented tools in the ED setting included posters for visual cueing, discussion in staff reporting and regular documentation of four factors embedded in the flowsheet (caregiver concern, unusual therapy, watcher patient, and communication breakdown).	Adapted from the Cincinnati Situational Awareness Model [2]
Escalation aid	The escalation aid outlines actions to support clinical decision making following assessment. Recommended mitigation actions (e.g. notification, reassessment, consultation) correspond to PEWS scores and situational awareness factors. A quick-view of the escalation aid was also embedded in the flowsheet.	Adapted from Cook Children’s Medical Center
Communication framework	The Situation, Background, Assessment, Recommendation (SBAR) toolkit was used to improve communication between team members regarding patient status.	SBAR toolkit [16]

**Fig. 1:**
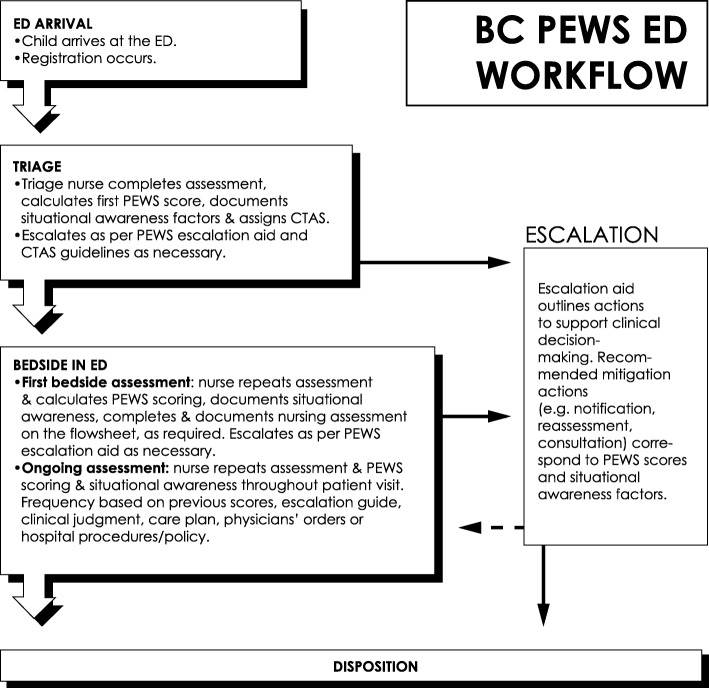
Flow map of PEWS in the Emergency Department. (CTAS: Canadian Triage and Acuity Scale)

PEWS scoring occurred at triage and then with each vital signs assessment throughout the stay. PEWS scoring was to be completed at a minimum of every 2 h for children with a PEWS score of 0–3 and more frequently for higher PEWS scores [4–13] (Fig. 1). We hypothesized that this system would enhance providers’ ability to recognize and communicate risk and accelerate mitigating actions.

### Intervention implementation and monitoring

A team consisting of staff from nursing, medicine, and administration assumed active site leadership roles in implementation and support to health practitioners. Educational resources that had been developed for the provincial inpatient implementation were used for training the nurses in this ED pilot, including instructions for using the flowsheet, e-learning training modules, on-site training workshops, and case studies. Training reinforced the concept that PEWS was a tool to aid clinical judgment, not replace it.

We monitored implementation fidelity through quality audits of medical records at five points during the one-year implementation. These audits were based on selected quality indicators: utilization rates of flowsheets, completion and accuracy of PEWS scoring, documentation of situational awareness factors, and escalation of care. Results were used to provide further education where needed and to promote optimal uptake.

### Evaluation

We took a mixed-methods approach, triangulating qualitative and quantitative data, to evaluate this intervention across the three dimensions of fidelity, effectiveness, and utility, following Campbell et al.’s framework for evaluation of complex health interventions [18]. These dimensions and their indicators were selected based on current literature on evaluation of PEWS initiatives, input from provincial stakeholders regarding relevance, and feasibility (Table 2).

**Table 2:** Dimensions, indicators, and sources of data selected for evaluation

Evaluation dimension	Indicators	Data source
Implementation fidelity: *whether the program was implemented as designed and staff were satisfied with it.*	PEWS score documentation	Post-implementation medical record eview
	Accuracy of PEWS score calculation	Post-implementation medical record review
	Satisfaction with PEWS in ED and its implementation	Online provider survey
	Barriers and facilitators in implementation	Online provider survey and key-informant interviews
Effectiveness: *whether the intervention changed practice.*	Increased vital signs documentation	Pre and post implementation medical record review
	Increased knowledge and confidence in pediatric care as a result of intervention	Online provider survey
	Perceived changes in practice as a result of intervention	Online provider surveys and key-informant interviews
	Improved communication between staff	Online provider survey
Utility: *whether the staff found the intervention valuable and it aligned with current practice.*	Alignment of PEWS score with CTAS	Post-implementation medical record review
	Perceived usefulness and value of PEWS in ED and its components in provision of care	Online provider surveys and key-informant interviews

### Data collection

We used three types of data collection techniques: medical record review, online survey of providers and semi-structured interviews with key informants to allow us to explore this system from multiple angles and offset the limitations or biases of any one method [19]. The medical record reviews allowed us to explore fidelity and feasibility of implementation, and the documented changes in practice. The qualitative methods allowed us to round out this information by answering questions related to what, why, how the system was working from the perspective of the ED providers [19].

#### Medical record review

We carried out retrospective medical record reviews covering the 12-months pre-implementation as baseline, and the 12 months post-implementation for comparison. This timeframe accounted for the full cycle of seasonal variation in ED presentations.

The inclusion criteria for medical record selection were patients who (i) were seen in ED in the 12 months pre and post implementation, and (ii) were under 17 years of age (the provincial age for defining a person receiving hospital care as “pediatric”), and (iii) had an ED length of stay greater than 2 h (to allow for trending), and (iv) had a disposition of either transfer to higher level of care or admission to hospital, and (v) had a CTAS assignment of 1–4 (as proxy measures of acuity and severity). We stratified our sample to include patients across all CTAS levels 1–4 and age groups (0–3 months, 4–11 months, 1–3 years, 4–6 years, 7–11 years, 12–17 years). The final sample included all patients transferred to a higher level of care, all CTAS 1 and 4 patients who were admitted to the hospital (as these were few in number) and 50% of the CTAS 2 and 3 patients who were admitted to the hospital, randomly selected within the stratified age groups.

Data were extracted from the medical records by two experienced pediatric acute care nurses and inputted in an electronic data capture tool hosted by the BC Children’s Hospital Research Institute. REDCap© (Research Electronic Data Capture) is a secure, web-based application designed to support data capture for research studies [20]. Identifiers were removed and a unique study code was generated for each patient. The reviewers maintained field notes, and in the case of uncertainty, decisions were made within the team.

#### Online survey of providers

One year post implementation, all ED nursing staff and physicians were invited to participate in an online survey to explore and describe key issues related to implementing PEWS in ED. Survey questions focused on exploring perceptions related to changes in practice, knowledge and confidence in providing pediatric care, usefulness of PEWS and its components, satisfaction with the tools and training, as well as barriers and facilitators to implementation. The survey took approximately 20 min to complete, included multiple choice questions, checkboxes, Likert scales, and open-ended questions, and was administered anonymously through Fluid Surveys, an online data collection platform. The survey was pilot tested by three experienced registered nurses prior to data collection and adjustments were made as needed.

#### Semi-structured interviews with key informants

The hospital administrator, registered nurse educator and physician who championed and supported the intervention were interviewed face-to-face in English for approximately 40 min after 12 months of implementation. Interview questions focused on understanding the utility of PEWS in ED and identifying the barriers and facilitators to implementation; this information was important for understanding how this complex system might function in a busy ED setting, to inform changes required and implementation processes. To confirm accuracy and credibility, the interviewer (YT) took notes capturing the responses and then summarized these for participants at the end of the interview.

### Quantitative data analysis

We used descriptive statistics to calculate the overall characteristics of patient medical records and Fisher’s exact test to determine if they were significantly different in any aspect. We used two-samples tests for equality of proportions to determine whether vital signs documentations significantly increased post-implementation compared to baseline. We used Spearman’s correlation to evaluate the relationship between PEWS scores and CTAS scores. Statistical analyses were carried out using Microsoft Excel and RStudio [21].

Response options of the online survey with 5-level Likert scales were collapsed into a measure consisting of 3 levels. Frequencies of responses were calculated for the different categories. In reporting survey responses, the denominators for the calculated percentages represent number of persons who responded to that particular question, not the whole survey.

### Qualitative data analysis

Qualitative data from open-ended survey questions and interviews with key informants were analyzed using thematic content analysis to identify the key elements of the responses [19]. Responses were reviewed, coded and categorized into commonly occurring themes by two separate members of the research team to enhance trustworthiness of the data. These researchers (TM and Research Associate JM-N) had experience and training with qualitative analysis in health research. The recurrent themes as identified by the researchers underwent member checking to ensure they accurately captured the perceptions of practitioners. This analysis method was chosen to address the primary aim of the qualitative methods: to explore and report on participant’s key perceptions as users of the new system. Saturation was obtained in the themes noted across the survey and interview responses.

## Results

### Collected data

In total, 192 medical records were selected for review; 96 at baseline and 96 at final evaluation. The two samples were not significantly different from each other (adopting a statistical significance of 5%), with the only exception being discharge disposition from ED, which can be explained by the opening of a pediatric short stay unit in the ED during the study period (Table 3).

**Table 3:** Characteristics of patients included in medical record review pre and post implementation (*n* = 96 for each sample)

Sample characteristic	Pre-implementation n (%)	Post-implementation n (%)	*p*-value *
Female / male	52 (54.2) / 44 (45.8)	39 (40.6) / 57 (59.4)	0.08
Age			
0–3 months	22 (22.9)	17 (17.7)	0.16
4–11 months	4 (4.2)	11 (11.5)	
1–3 years	20 (20.8)	23 (24.0)	
4–6 years	14 (14.6)	18 (18.8)	
7–11 years	16 (16.7)	17 (17.7)	
12–16.9 years	20 (20.8)	10 (10.4)	
Most responsible diagnosis ^a^			
Respiratory	23 (24.0)	26 (27.1)	0.24
Gastrointestinal	10 (10.4)	19 (19.8)	
Hyperbilirubinemia	10 (10.4)	8 (8.3)	
Other diagnosis	53 (55.2)	43 (44.8)	
Discharge disposition from ED			
Transferred to higher level of care	37 (38.5)	22 (22.9)	0.03
Admitted internally	59 (61.5)	74 (77.1)	
CTAS level			
CTAS 1	7 (7.3)	10 (10.4)	0.05
CTAS 2	44 (45.8)	43 (44.8)	
CTAS 3	40 (41.7)	28 (29.2)	
CTAS 4	5 (5.2)	15 (15.6)	

*CTAS* Canadian Triage and Acuity Scale, *ED* Emergency Department, *PEWS* Pediatric Early Warning System

* *P*-value calculated with Fisher’s exact test for count data

^a^ 3% entries missing during pre-implementation review. Diagnosis based on most affected system. If multiple diagnoses were presented in discharge summary, priority was given to the first one written

As for provider surveys, the response rate was 38% (*n* = 37) for registered nurses and 72% (*n* = 13) for physicians. Half of the practitioners had less than 5 years of clinical work experience. 39% (*n* = 5) of physicians and 21% (*n* = 8) of nurses reported having more than 15 years of work experience. Most estimated that 10–25% of their practice was pediatric care.

Three key-informant interviews were conducted.

### Implementation fidelity

Medical record reviews show that the intervention was implemented with high fidelity. At triage, 80.2% (*n* = 77) of the medical records had a PEWS score completed and of those 87% (*n* = 67) had been accurately calculated. At first bedside assessment, 81.2% (*n* = 78) of the medical records had a PEWS score present and of those 88.5% (*n* = 69) were accurate. Medical record review found the most common reason for inaccuracy in PEWS score to be adding the sub-scores instead of taking the highest score for the section, a matter that key informants suggested was improved through practice and more education. At both triage and first bedside assessment, highly urgent or non-urgent patients (CTAS scores 1 and 4) were more likely to be missing a PEWS score than others (*p*-value = 0.0375 for triage and 0.0354 for bedside assessment, Fisher’s exact test). In 52.1% (*n* = 25) of patients with an initial PEWS score of 0–3, the time in between assessments was 2 h or less, as per the implementation plan. In 61.4% (*n* = 59) of the records, a PEWS score was completed at all vital signs assessments throughout the ED visit. In an additional 29.2% (*n* = 28), PEWS score was completed for over half of the vital signs assessments.

Overall, majority of survey respondents were satisfied or very satisfied with PEWS scoring system (71.8% of nurses, *n* = 23; 81.8% of physicians, *n* = 9), PEWS flowsheet (56.2% of nurses, *n* = 18; 81.8% of physicians, *n* = 9), escalation guide (68.8% of nurses, *n* = 22; 81.8% of physicians, *n* = 9), and reference cards (75% of nurses, *n* = 24; 70% of physicians, *n* = 7). Satisfaction was relatively lower for situational awareness tools (41.9% of nurses, *n* = 13; 36.4% of physicians, *n* = 4) and the communication framework (54.5% of nurses, *n* = 17; 45.5% of physicians, *n* = 5).

### Intervention effectiveness

Comparison of medical records from before and after implementation showed that adding PEWS scoring to the assessment of pediatric patients significantly increased the rates of documentation of seven parameters embedded in the score at first bedside assessment and throughout the ED stay (Table 4).

**Table 4:** Completeness of documentation based on medical record review pre and post implementation

PEWS score component	Pre-implementation (*n* = 96)	Post-implementation (*n* = 96)	Increase	*p*-value *
Documentation of parameters at first assessment in the ED				
Respiratory rate	60 (62.5)	94 (97.9)	57%	< 0.01
Oxygen concentration	62 (64.6)	90 (93.8)	45%	< 0.01
Respiratory distress	53 (55.2)	88 (91.7)	66%	< 0.01
Heart rate	63 (65.6)	94 (97.9)	49%	< 0.01
Capillary refill time	29 (30.2)	86 (89.6)	> 100%	< 0.01
Skin colour	41 (42.7)	86 (89.6)	> 100%	< 0.01
Behaviour	56 (58.3)	91 (94.8)	63%	< 0.01
Average	52 (54.2)	90 (93.6)	84%	< 0.01
Consistent documentation of parameters throughout ED stay ^a^				
Respiratory rate	30 (31.3)	91 (94.8)	> 100%	< 0.01
Oxygen concentration	28 (29.2)	83 (86.5)	> 100%	< 0.01
Respiratory distress	6 (6.3)	80 (83.3)	> 100%	< 0.01
Heart rate	32 (33.3)	94 (97.9)	> 100%	< 0.01
Capillary refill time	0 (0.0)	81 (84.4)	–	< 0.01
Skin colour	1 (1.0)	82 (85.4)	> 100%	< 0.01
Behaviour	5 (5.2)	82 (5.4)	> 100%	< 0.01
Average	15 (1.2)	85 (88.2)	> 100%	< 0.01

Note: Percentages are shown in parenthesis

* *p*-value calculated with two-sample test for equality of proportions

^a^ Consistent documentation refers to documentation of each parameter with every assessment

Using PEWS in ED further promoted documentation of two situational awareness factors throughout the patient stay: recording of “caregiver concern” increased from 10.4 to 67.7%, and “watcher patient” from 2.1 to 40.6%. “Watcher patient” was noted by the chart reviewer to be particularly important for documenting risks that PEWS score is not designed to capture, including: pain, surgical risk, abnormal lab values, neurologic status, and mental health risk.

We also observed a 51% increase in documentation of notification of most responsible physician and documentation of response by physician.

The majority of practitioners responding to the post-implementation survey reported that the implementation of PEWS in ED changed their knowledge of and confidence in providing pediatric care to a “great” or “very great” extent. The biggest improvement in knowledge was in identification of abnormal clinical signs (58.1%, *n* = 25), change to a great or a very great extent (Table 5).

**Table 5:** Intervention effectiveness: perception of change in knowledge and confidence of practitioners

Perceived change in knowledge and confidence	Not at all /to a slight extent (%)	To a moderate extent (%)	To a great / very great extent (%)
Change in knowledge			
Identification of abnormal clinical signs	25.6	16.3	58.1
Identification of situational awareness factors that increase risk	34.9	20.9	44.2
Mitigation of deterioration	23.3	34.9	41.9
Escalation of care	25.6	25.6	48.8
Change in confidence			
Identification of abnormal clinical signs	23.3	34.9	41.9
Identification of situational awareness factors that increase risk	32.6	37.2	30.2
Mitigation of deterioration	30.2	32.6	37.2
Escalation of care	32.6	30.2	37.2

Interviewed key-informants highlighted a few important issues about the effects of implementing PEWS at triage: 1) the tool was easily accepted and used, i.e. its adoption took little promotion or management from leadership, 2) time to complete PEWS scoring decreased with practice, and 3) the slight increase in triage time was outweighed by thoroughness of assessment and greater awareness of patient status; this was particularly important when the department was busy and wait times were long. Surveyed physicians noted an overall increase in staff awareness regarding pediatric patients, which promoted earlier notification. *“I think the general increase in awareness of frontline staff has improved, and that either escalates the CTAS score, or prompts the nurses and others to bring the patient to our attention sooner”* (Physician).

Lastly, the survey respondents reported enhanced communication between practitioners with the implementation of PEWS, particularly regarding timing of verbal communication but also frequency and clarity (Table 6). One nurse highlighted: *“If the PEWS score is rising or falling, it gives us a reason to contact the MD in clear, concise language”*.

**Table 6:** Implementation effectiveness: perception of improvement in verbal communication attributed to PEWS in ED

Perceived improved verbal communication between practitioners	No (%)	Somewhat (%)	Yes (%)
Frequency of verbal communication	17.9	30.8	51.3
Timing of verbal communication	20.5	20.5	59.0
Clarity of verbal communication	23.1	25.6	51.3
Outcomes of verbal communication	17.9	35.9	46.2

### Intervention utility

Overall, the majority of survey respondents (78.9%, *n* = 30) agreed that implementation of PEWS in the ED was valuable for pediatric patient care. One nurse highlighted: *“It increased our response to children with abnormal vital signs that are compensating or appear relatively well.”* The leadership also noted: “*PEWS helped validate their clinical decision making and offered a standardized approach to care.*”

The PEWS score, assessment flowsheet, and escalation were overall seen as highly useful (average 78.3% indicated useful to moderate extent or higher). While the situational awareness and communication framework were not as favorable, they were still seen as moderately useful or higher by over 65% of the respondents (Table 7).

**Table 7:** Intervention utility: perception of usefulness of different BC PEWS components

Perceived usefulness	Not at all / to a slight extent (%)	To a moderate extent (%)	To a great / very great extent (%)
Pediatric assessment flowsheet	25.6	32.6	41.9
PEWS score	20.9	23.3	55.8
Situational awareness ^a^	38.0	31.0	31.0
Escalation aid	18.6	27.9	53.5
Communication framework	30.2	32.6	37.2
Overall value to pediatric care in ED	5.3	15.8	78.9

^a^ Average of responses of the four situational awareness factors (caregiver concern, unusual therapy, watcher patient and communication breakdown)

Some participants perceived little value in using PEWS (5.3%, *n* = 2). Their reasoning related to the need to further tailor the flowsheets to the ED environment: “*PEWS form is good, but frustrating to have to use old form too. It would be great if there was an ED specific PEWS form”* (Nurse).

To study alignment of the intervention with current practice, we explored how the PEWS score relates to CTAS score, the well-established triage scoring system. Seventy-seven charts included both a CTAS score and PEWS score at triage. Collapsing PEWS scores 5–13 into a single score (since they would trigger the same escalation guide), we found PEWS scores (0–5) and CTAS scores [1–4] to be inversely correlated (Spearman’s *rho* = − 0.574, *p*-value < 0.001).

The majority of surveyed nurses (89.6%, *n* = 25) felt that it was “valuable” or “possibly valuable” to complete a PEWS score at triage alongside CTAS (physicians are not involved in triage, therefore were not asked these questions). Practitioners noted that the PEWS score provided a baseline for trending across the visit and gave more objective, thorough results by promoting assessment of all parameters consistently with all patients, which helped to correctly assign a CTAS score. *“It (PEWS score) helps me a lot with assessment and priority settings in triage especially for the pediatric population who came in really sick and unable to obtain complete information/data from parents, guardians or significant other”* (Nurse).

The impressions of staff regarding the impact of PEWS in ED on pediatric practice are summarized in Table 8. Matters such as earlier identification of risk, more comprehensive assessment, and standardized approaches to communication and mitigation were seen as positive changes. Meanwhile, scores not representing the degree of risk were a reported weakness, particularly at triage e.g. false positive scores when the child is upset or crying, has fever or has been given medication for symptom relief: *“Many children find triage to be overwhelming and often are crying, increasing their PEWS score despite looking well”* (Nurse).

**Table 8:** Themes of perceived positive and negative impacts of PEWS in ED on pediatric practice

Themes	Sub-themes	Quotes
Perceived positive impacts		
Identification	• Prompts earlier recognition of risk, change, decline, and abnormality • Increases provider’s general awareness of risk, concern, and abnormality • Guides triage decisions	*“With the PEWS scores that I gathered after assessment, it helped me alert the primary nurse and emergency doctors to see patient as soon as possible and be able to render care in a timely manner.”- Nurse*
Assessment	• Provides a standardized assessment framework • Improves ease and comprehensiveness of assessment (full vital signs) • Increases staff comfort with vital signs norms	*“Caring for a pediatric patient can be very stressful for some nurses, the flowsheet helps guide the inexperienced nurse through a very thorough assessment.”- Nurse*
Monitoring	• Provides a baseline for monitoring from triage onwards • Increases frequency of vital signs assessment and closer observation • Improves ability to trend across patient stay which helps with care and disposition decisions	*“Children get vitals more often and all of their vitals more often! It is easy to see what vitals are normal and what are not with graphs and colours. It is easy to see changes and trends in vitals. It likely prompts more repeat vitals and assessments and documentation thereof. When done at triage prompts full vitals early.”- Physician*
Communication	• Provides a standardized approach to communication (speaking the same language) • Promotes earlier notification of physicians • Enhances delivery of thorough information to physicians • Improves confidence of nurses with notification (validation by score)	*“Effective as a means of communicating with doctors, helpful in confirming if patient is improving as appearance can be deceiving, PEWS assists with confirmation.”- Nurse*
Mitigation	• Provides a standardized approach to escalation • Supports earlier response as notification occurs faster	*“I think it is very important data when it comes to assessment, priority setting and rendering time sensitive actions to every peds patient.”- Nurse*
Other	• Promotes better overall care for pediatric patients	*“PEWS has really played a huge role in the way in which we care for and treat our pediatric patients. This has been a real positive change.”-Nurse*
Perceived negative impacts		
Accuracy	• Scores may not accurately capture clinical status in some instances (e.g. false positive scores due to upset, post medication)	*“Need to alert staff that PEWS can be elevated due to patient stress in ED environment/circumstances, crying, tantrum,* etc. *Thus, require a strategy for these circumstances* i.e. *using clinical judgment, repeating scoring when patient calms.” - Nurse Educator*
Autonomy	• Standardized scoring and escalation can take away from clinical judgement	*“In my experience as charge nurse, we use clinical judgment, vital signs anyways, and the PEWS is just a numerical value of our own clinical assessment.” - Nurse*
Workload	• Can increase time for assessment (particularly at triage)	*“Added paperwork and time spent especially at triage” - Nurse*
Lack of tailoring to ED setting	• Increases paperwork because poorly integrated with current ED paperwork (double charting) • Form is missing important information for ED (e.g. narrative space, medication record)	*“Increased charting as not everything can be charted on one paper” - Nurse*
Relevance	• Lacks relevance or seems excessive for patients with single system or minor injuries	*“It seems too time consuming and unnecessary to do for all children presenting in the ED. There are a lot of very simple presentations that do not seem to warrant the full set of vitals and score. For example, children coming in with very small lacerations or simple wounds, or even simple injuries.” - Nurse*

## Discussion

This study presents the implementation of PEWS in the ED of a general urban hospital in British Columbia, Canada. Employing a framework for evaluation of complex healthcare interventions and using medical record reviews, surveys, and key-informant interviews, we evaluated the intervention’s fidelity, effectiveness, and utility [18]. Our study supports that a multi-faceted PEWS can be valuable in supporting ED practitioners with providing timely and effective pediatric care. However, we concluded that it is important that the tools and training strategy are tailored to the ED context to enhance implementation fidelity and satisfaction.

### Using PEWS and CTAS at triage

In this pilot intervention, PEWS scoring was completed at triage alongside CTAS scoring [14]. These two scores have been designed for different purposes and their scoring is done differently: PEWS identifies early signs of deterioration and is scored through assessment of vital signs [4], while CTAS assigns acuity and is scored via assessment and clinical judgement [14]. We were able to show that the scores correlate and that practitioners found them synergistic; we therefore conclude that there is benefit in using both systems with pediatric patients. The PEWS score calculation reinforces a more comprehensive, objective assessment informed by vital signs norms based on age. In our study, implementation of PEWS significantly increased documentation of physiologic parameters; a benefit reported by others [1]. This systematically addresses issues of variance across providers and as highlighted by Roland et al. [7], may reduce cognitive bias, errors or missed assessment components that could otherwise occur because of the pressures of the triage environment [22].

Study participants noted a few limitations when using PEWS at triage. An increase in time at triage was highlighted, an issue which improved with practice and was largely acceptable to practitioners who indicated that the value of scoring outweighed time increase. There were also instances of false positive scores owing to factors such as irritability, which altered vital signs and skewed PEWS scores. In our study, we aimed to counteract this issue through reinforcing the importance of clinical judgement and repetition of assessment when score accuracy was in question. Limited specificity has also been reported elsewhere [9, 10, 12]. As parameters included in a PEWS score assessment are important for determining risk, this substantive increase in thorough assessment and documentation at triage is a very positive change. The limitations of PEWS were acceptable to practitioners who overall found value in using PEWS at triage alongside the CTAS triage tool.

### Using the 5-component BC PEWS at bedside evaluation

It has been demonstrated that nurses and physicians may fail to recognize deteriorating children due to lack of consistency or accuracy in recording physiological observations [23, 24]. Consistent and comprehensive documentation has been shown to be particularly important in the ED environment where health care providers need to document improvement and appropriateness for discharge [7]. Moreover, with pediatric patients, this documentation is even more critical because providers often rely on general appearance and vital signs as opposed to patients’ descriptions of improvement. This study reviewed documentation of PEWS components across the patient stay. The introduction of PEWS was associated with a substantive increase in documentation across all physiologic parameters. We conclude that the introduction of PEWS in ED can have a positive influence on rates and thoroughness of documented assessment parameters. This not only provides a more consistent “in the moment” picture of physiologic abnormality, but also allows for documentation of trending from the point of presentation.

### The “active ingredients” of BC PEWS

BC PEWS was designed as a multifaceted safety system as advocated by Lambert et al. [4]. In trying to understand its utility as a system, we asked practitioners to assess the usefulness of each of the components. While all of the components of BC PEWS were ranked useful by the majority of respondents, the PEWS escalation aid and score were perceived as most useful. Practitioners reported that the norm-based scoring enhanced or validated identification of sick children [7]. The escalation aid justified escalating to senior review and encouraged repeated assessment or more thorough monitoring throughout the patient stay.

The systematic documentation and reporting of situational awareness factors were ranked as the least useful part of the system. Although there was a substantive rise in the documentation of “caregiver concern” and “watcher patient”, the medical records reviewer indicated instances where “watcher patient” was missed as an opportunity to elevate a risk profile. For instance, as has been shown in the literature, PEWS scores are more accurate in capturing significant medical illness (particularly respiratory related) than surgical risk [11]. Medical record reviews noted instances such as surgical risk, mental health risk, abnormal labs or neurovitals, where “watcher patient” would have been an appropriate and systematic means of elevating risk profile and guiding appropriate action. Thus, despite lower usefulness rankings, we believe that situational awareness factors highlight risk and create awareness beyond scoring, which has value as part of a safety system in the ED. Moving forward, we believe more tailoring to ED context and further education of staff may improve uptake. Context and staff perceptions play crucial roles in PEWS implementation success as we and others have observed [25, 26]. In addition, as current research focuses on the inpatient setting, further investigation of the impact of situational awareness in the ED on care and patient outcomes will be important.

### Communication within the team

Enhanced communication is a demonstrated factor in improving quality healthcare [16]. Communication breakdown at handovers are a known point of risk that require consideration when planning an effective system [7]. Because BC PEWS was implemented across the province in inpatient settings, our team chose to implement the same tools to promote a common language, understanding and process across sites and departments, and within the ED. Our findings support that this implementation strategy was effective in enhancing communication as our survey found positive influence on verbal communication and our medical records review found significant gains in documentation. Further, staff reported increased comfort with escalation and communication of risk for senior review, which is an important characteristic of an optimal PEWS tool. As noted above, the staff attributed this gain to having objective tools to validate the need and process for escalation [25]. While we will be taking feedback from this research to revise an assessment flowsheet for ED, we found value in a consistent scoring tool to promote internal and external communication and will retain the Brighton [15] tool in scale up.

### PEWS, knowledge and confidence through standardization

As described by Oldroyd and Day, there is a lack of exposure to seriously ill children among ED nurses in general hospitals [8]. Low practice volumes present challenges with maintenance of competency, thus guidance provided by PEWS can assist with identification of serious illness [8]. Our study showed that nurses reported improvements in knowledge and confidence post-PEWS implementation and were completing more comprehensive assessment of vital signs that improved their ability to trend the patient stay. Physician respondents indicated similar benefits in knowledge and confidence and site leaders expressed an overall perception that the healthcare team showed greater awareness of risk or abnormality in the pediatric population. Importantly, improvement in staff knowledge and confidence for providing provision of pediatric care has been noted to be one of the key outcomes of PEWS implementation, which unlike patient outcomes is robustly measurable [27].

Given the decrease in variability of assessment practices using BC PEWS, we conclude that the implementation of PEWS influenced the equalization of patient care between providers. Additionally, having vital signs reference ranges visually evident in PEWS decreased knowledge deficit and cognitive load that may be present for staff who are less familiar or experienced with the pediatric population.

A minority of survey respondents perceived that systems such as PEWS take away from autonomy of experienced nurses. While overall findings of the study support positive associations with the use of such standardized systems, this finding suggests a need to acknowledge and reinforce the role of such systems in supporting clinical judgement rather than replacing or usurping it [26, 28].

### The overall value of PEWS for ED

Bonafide et al. demonstrated that beyond the marginal performance of PEWS when applied to data sets, clinicians who recently experienced PEWS score failures (false positives) still considered it valuable [29]. In a survey of 254 general ED practitioners in the UK, Griffiths and Kidney found that the majority support the use of early warning systems in the ED, despite the evidence that such scores are low in sensitivity [30]. As with other studies, we found limitations with PEWS in the ED; however these limitations did not appear to alter the overall value assigned to PEWS by staff.

Roland et al. outlined that early warning scores in ED should support earlier diagnosis, more accurate estimation of illness severity and improved communication [7]. Our participants reported that BC PEWS fulfilled all of these functions.

An effective clinical tool is one that practitioners use successfully, and one they want to use [25, 26]. Notably, the hospital team continued to use BC PEWS after the conclusion of the study and have provided ongoing support to the re-design of the system and scale up for the province.

### Informing scale up

This study demonstrated that BC PEWS is useful and beneficial to pediatric patient care when used with clinical judgment and alongside the currently accepted tools (CTAS) in the ED.

Based on these results and the growing body of evidence in the literature, provincial stakeholders made the unanimous decision to move to provincial implementation of PEWS in all ED settings in British Columbia. This pilot study’s results were used to redesign the 5-component BC PEWS specifically to address the needs of EDs (BC PEWS ED). This involved the creation of a provincial emergency nursing assessment record (short and long form versions) with a compatible PEWS scoring sheet for recording vital signs, and a provincial escalation guide for the ED, which corresponds to PEWS scores. Training materials were developed for ED staff including online training modules, in-person workshops, and a series of quick educational sessions that can be offered as refreshers. Health Authorities from across the province reviewed and accepted the new system to ensure buy-in and regional support. Beginning in February 2018, the province launched a phased implementation of BC PEWS ED, which included the full range of EDs serving children from the generalist rural/remote facilities to the provincial subspecialty pediatric facility. The implementation is being monitored through quarterly audits and planning is underway for researching the scale up of implementation.

### Study limitations

The study has a number of limitations. First, there were no accurate measures of deterioration available when identifying the dataset for review so proxy measures of deterioration or severity (i.e. transfer to higher level of care, admission, triage score) were used to select medical records for review. However, we believe that the selected proxy measures captured the population of interest. Second, response rate for the health providers survey was limited to 55%, even after sending out reminders in an attempt to increase the rate. However, our response rate is high compared to a study looking at response rates to online surveys among Canadian physician specialists [31]. Nevertheless, a non-response bias cannot be ruled out. The study did not account for an acclimatization phase in the implementation and did not look at impact on patient outcomes, matters that will be addressed during the research of the provincial scale up.

## Conclusion

In this study, standardization of pediatric ED care through PEWS demonstrated several benefits: PEWS significantly improved comprehensiveness of assessment and documentation at triage and throughout the ED stay. Accordingly, there was a perception that identification and awareness of risk rose and equalized between practitioners. BC PEWS upgraded providers perceived knowledge and confidence related to pediatric care, and increased timely and effective communication between practitioners. Scoring and the escalation aid made it objectively clear when senior review was required, helping to remove ambiguity and variance. Consequently, we believe that BC PEWS contributed to addressing the issues of cognitive load seen in typical ED settings, particularly amongst practitioners for whom pediatrics is a small part of a heavy caseload. Finally, all components of the system were perceived and reported as useful, and their use continued and evolved beyond the end of the study period, most importantly in planning for provincial scale up.

## Data Availability

Any datasets not included in this published article are available from the corresponding author on reasonable request.

## Abbreviations

BC: British Columbia
CTAS: Canadian Triage and Acuity Scale
ED: Emergency Department
PEWS: Pediatric Early Warning System
SBAR: Situation, Background, Assessment, Recommendation
